# Methods for Estimating Demography and Detecting Between-Locus Differences in the Effective Population Size and Mutation Rate

**DOI:** 10.1093/molbev/msy212

**Published:** 2018-11-14

**Authors:** Kai Zeng, Benjamin C Jackson, Henry J Barton

**Affiliations:** 1 Department of Animal and Plant Sciences, University of Sheffield, Sheffield, United Kingdom; 2 Institute of Evolutionary Biology, School of Biological Sciences, University of Edinburgh, Edinburgh, United Kingdom

**Keywords:** inferring demography, effective population size, mutation rate, comparing sex chromosomes and autosomes, sex ratio evolution

## Abstract

It is known that the effective population size (*N*_e_) and the mutation rate (*u*) vary across the genome. Here, we show that ignoring this heterogeneity may lead to biased estimates of past demography. To solve the problem, we develop new methods for jointly inferring past changes in population size and detecting variation in *N*_e_ and *u* between loci. These methods rely on either polymorphism data alone or both polymorphism and divergence data. In addition to inferring demography, we can use the methods to study a variety of questions: 1) comparing sex chromosomes with autosomes (for finding evidence for male-driven evolution, an unequal sex ratio, or sex-biased demographic changes) and 2) analyzing multilocus data from within autosomes or sex chromosomes (for studying determinants of variability in *N*_e_ and *u*). Simulations suggest that the methods can provide accurate parameter estimates and have substantial statistical power for detecting difference in *N*_e_ and *u*. As an example, we use the methods to analyze a polymorphism data set from *Drosophila simulans*. We find clear evidence for rapid population expansion. The results also indicate that the autosomes have a higher mutation rate than the X chromosome and that the sex ratio is probably female-biased. The new methods have been implemented in a user-friendly package.

## Introduction

Information on past demographic changes is essential for understanding how major events shape the evolution of a species (e.g., the out-of-Africa migration of humans; Veeramah and Hammer 2014), for reliably detecting genes underlying adaptation/speciation (Bank et al. 2014; Payseur and Rieseberg 2016), and for formulating effective conservation strategies (Allendorf et al. 2010). As a result, many methods have been developed for making demographic inferences by examining various aspects of sequence polymorphism (Schraiber and Akey 2015; Payseur and Rieseberg 2016).

Due to the randomness of the process of evolution and the rarity of polymorphic sites, the amount of information provided by data from a small genomic region is rather limited, which in turn leads to large statistical noise in the inference. This problem is typically dealt with by combining data from multiple loci. However, this approach is complicated by regional heterogeneity in important parameters. For instance, the mutation rate, *u*, varies across the genome (Hodgkinson and Eyre-Walker 2011). In addition, the effective population size, *N*_e_, is also heterogeneous (*N*_e_ is inversely related to the rate of coalescence; Charlesworth 2009). Variation in *N*_e_ may be caused by differences in the mode of inheritance (e.g., autosomes vs. sex chromosomes; Charlesworth 2009) and/or differences in the strength of selection at linked sites (e.g., selective sweeps and background selection; Cutter and Payseur 2013).

To illustrate problems with combining data from multiple loci, imagine that there was a 10-fold increase in the population size 1,000 generations ago and that we have data from two loci with effective population sizes 5,000 and 100, respectively. It is well known that the level of polymorphism is determined by $\theta =4N_{e}u$. Thus, if *u* is the same at the two loci, locus 1 is expected to contribute more SNPs to the combined data set due to its higher *N*_e_. On the other hand, when expressed in units of two times the locus-specific *N*_e_, the scaled time to the expansion event is 0.1 for locus 1 and 5 for locus 2. Thus, locus 1 is much closer to the event than locus 2, and its local genealogy is expected to deviate more from that expected under an equilibrium model. As locus 1 makes a larger contribution to the combined data set, making inferences on the combined data without regard to these between-locus differences will lead to results that are biased toward the situation at locus 1.

Being able to detect differences in *N*_e_ and *u* between loci is required for studying important questions in evolution. For instance, comparing sex chromosomes and autosomes with regard to their polymorphism patterns is a powerful way of detecting evidence for an unequal sex ratio and/or sex-biased demographic processes (Webster and Wilson Sayres 2016). Although existing methods developed for this purpose do take into account variation in *N*_e_ and *u* between sex chromosomes and autosomes (Pool and Nielsen 2007, 2008; Garrigan 2009; Keinan et al. 2009; Haddrill et al. 2011; Evans et al. 2014; Clemente et al. 2018), they are limited in several important aspects: 1) Some rely on summary statistics such as the X-autosome diversity ratio, and do not make full use of the data (Pool and Nielsen 2008); 2) some cannot detect changes in the sex ratio between different evolutionary epochs (Garrigan 2009; Haddrill et al. 2011; Evans et al. 2014); and 3) some do not model the mutation process, and therefore cannot detect difference in the mutation rate between sex chromosomes and autosomes caused by, for example, male-driven evolution (Clemente et al. 2018).

Demographic inference methods concerned with data collected from within autosomes (or sex chromosomes) seem to pay less attention to regional variation in *N*_e_ and *u* (Gutenkunst et al. 2009; Excoffier et al. 2013). The method of Bhaskar et al. (2015) allows *u* to vary across loci but assumes a single *N*_e_ for all loci. The method of Gossmann et al. (2011) considers between-locus differences in both *N*_e_ and *u* but assumes that the population size is constant over time. Beaumont and colleagues (Beaumont 1999; Storz and Beaumont 2002) developed a hierarchical Bayesian model that accommodates changes in population size as well as variation in both *N*_e_ and *u*. However, this method is applicable to microsatellite data only. Finally, the method of Hey and colleagues (Hey and Nielsen 2004; Sousa et al. 2013) considers both demography and between-locus differences but is computationally intensive and not suitable for analyzing data sets with many loci.

To solve the issues discussed above, we describe a general framework for simultaneously inferring past changes in population size and detecting variation in *N*_e_ and *u*. Several methods are constructed, either for making comparisons between the X (or Z) chromosome and autosomes or for analyzing multilocus data from within autosomes (or sex chromosomes). The methods typically make inferences on polymorphism data, although some of them are able to use both polymorphism and divergence data. Using computer simulations, we ask the following questions: 1) To what extent do regional differences in *N*_e_ and *u* bias results obtained by demographic inference methods that ignore this heterogeneity? 2) Can the new methods overcome these biases? 3) Do the new methods have sufficient statistical power for detecting between-locus differences in *N*_e_ and *u*? As an example, we use the methods to analyze a polymorphism data set from *Drosophila simulans* (Jackson et al. 2017), focusing on X-autosome comparisons. We examine whether the population size has changed recently, whether *u* differs between the X chromosome and the autosomes, and whether there is evidence for sex-biased processes (e.g., an unequal sex ratio, sex differences in reproductive success).

## New Approach

### The General Model without Divergence Data

Consider a randomly mating diploid population. Going backward from the present, the population size changes in a stepwise manner with *H* epochs (see supplementary table S1, Supplementary Material online, for a list of mathematical symbols). The most recent epoch is referred to as epoch 1, the next epoch as epoch 2, and so forth. It is assumed that epoch *H* (i.e., the most distant epoch) extends indefinitely into the past, whereas the duration of epoch *h* is *T_h_* generations (1≤h<H). Let us focus on a locus in the genome, referred to as locus 1. It is assumed that the *N*_e_ at this locus in epoch *H* is *N*_1_. The population size during epoch *h* is $g_{1,h}N_{1}$ (1≤h<H). Mutation is modeled by the infinite-sites model. Let *u*_1_ be the mutation rate per site per generation. We define the scaled mutation rate as $\theta_{1}=4N_{1}u_{1}$, and the scaled time as $\tau_{h}=T_{h}/(2N_{1})$ (1≤h<H).

Consider a second locus, referred to as locus 2. Because the underlying demographic process is shared by all loci in the genome, the timing of population size changes (i.e., the *T_h_*s) is the same across loci (see supplementary fig. S1, Supplementary Material online, for a graphical representation of the model and its parameters). To model the difference in *N*_e_ between locus 1 and 2, we treat locus 1 as “the reference locus” and assume that the local *N*_e_ at locus 2 in the most distant epoch (i.e., epoch *H*) is $N_{2}=f_{2}N_{1}$. To model variation in the mutation rate, we assume that the mutation rate at locus 2 is *u*_2_ per site per generation and define the scaled mutation rate as $\theta_{2}=4N_{1}u_{2}$ (note that all scaled parameters are defined with respect to the reference locus). Finally, to accommodate the possibility that these two loci may respond differently to the demographic changes (e.g., sex-biased demographic processes can affect X-linked and autosomal loci differently; Webster and Wilson Sayres 2016), the population size during epoch *h* is assumed to be $g_{2,h}N_{2}=g_{2,h}f_{2}N_{1}$ (1≤h<H).

More generally, with data from *K* loci, the model has the following parameters, denoted collectively by Θ: 1) the time parameters τ = (*τ*_1_, *τ*_2_,…, $\tau_{H-1}$), which are shared across loci; and 2) the locus-specific parameters *θ_k_*, *f_k_*, and $g_{k}$ = ($g_{k,1},\;g_{k,2}$,…, $g_{k,H-1}$) (1≤k≤K). Note that *f*_1_ is fixed at 1 for identifiability of the parameters. Under this parameterization, information on variation in local *N*_e_ is provided by the *f_k_*s. Because the *θ_k_*s are defined with respect to *N*_1_, they are directly comparable between loci and provide information about variability in the mutation rate. By checking whether $g_{i,h}/g_{j,h}$ differs significantly from 1, we can examine whether locus *i* and *j* respond differently to demographic changes. It should be noted that the parameters are identifiable if and only if there have been recent changes in population size. In contrast, if the population size is constant (i.e., *H *=* *1), then polymorphism patterns at the *K* loci are fully characterized by the composite parameter $\theta_{k}^{*}$, defined as $4N_{1}f_{k}u_{k}$ (1≤k≤K).

Without loss of generality, we assume that samples of *n* alleles have been obtained from all *K* loci. The data from locus *k* are summarized using the unfolded site-frequency spectrum (uSFS), denoted by $d_{k}$ = ($d_{k,1},\;d_{k,2}$,…, $d_{k,n-1}$), where $d_{k,i}$ is the observed number of segregating sites of derived allele frequency *i*. Let d = ($d_{1},\;d_{2}$,…, $d_{K}$) denote all the data. As detailed in Materials and Methods, we calculate the (composite) likelihood of the data using the Poisson random field model (Sawyer and Hartl 1992; Bustamante et al. 2001), assuming neutrality, free recombination between sites, and the infinite-sites model of mutation. This allows us to obtain maximum likelihood estimates (MLEs) of the parameters (see Materials and Methods).

### The General Model with Divergence Data

We seek to increase the statistical power of the above model by appealing to the fact that the level of divergence to an outgroup species carries information about the mutation rate. For simplicity, we consider divergence between a sequence from the ingroup species and a sequence from the outgroup. Here it is important to consider the effects of ancestral polymorphism, which may account for a substantial fraction of the divergence level between closely related species (e.g., ancestral polymorphism may account for more than 24% of divergence between humans and chimpanzees; Chen and Li 2001).

Consider locus *k*. It is assumed that *N*_e_ at this locus in the population of the ancestral species is $cf_{k}N_{1}$, where the parameter *c* is used to model the possibility that the ancestral population is of a different size (recall that locus 1 is used as the reference locus and $f_{1}=1$). The expected divergence level is $\lambda_{k}=m_{k}(4cf_{k}N_{1}u_{k}+2t^{*}u_{k})$, where *m_k_* is the length of locus *k* in basepairs, and $t^{*}$ is the divergence time in generations. The first term in the parentheses describes differences accumulated within the ancestral population, and the second term considers changes accrued after speciation. We can rewrite *λ_k_* as $m_{k}\theta_{k}(cf_{k}+t)$, where $t=t^{*}/(2N_{1})$. Thus, the inclusion of divergence data introduces two new parameters, *c* and *t*, which are shared across loci. Let X = (*x*_1_, *x*_2_,…, *x_K_*) where *x_k_* is the observed number of substitutions at locus *k*. The data set now includes both X and d, and the new model has parameters Θ, *c*, and *t*. The likelihood of X can be calculated by assuming that the number of substitutions follows a Poisson distribution with mean *λ_k_*, as in previous studies (Gossmann et al. 2011; Galtier 2016; Tataru et al. 2017). This is then combined with the likelihood of d to obtain the overall likelihood (see eq. 9). It should be noted that the information on *c* and *t* comes from variation in divergence level across loci. Thus, this model should not be used to analyze data sets containing a small number of loci (see Results for more detail).

### A Simplified Model

The models described above are general in that they allow each locus to have its private parameters (i.e., *θ_k_*, *f_k_*, and $g_{k}$). They are parameter-rich and require each locus to be sufficiently large so that enough information is available for estimating the locus-specific parameters. Thus, the general model is more suitable for analyzing large genomic regions (e.g., the X chromosome vs. autosomes). Regarding data collected from multiple autosomal (or sex-linked) loci, it is reasonable to define a simplified model with $g_{h}=g_{k,h}$ (1≤k≤K and 1≤h<H). That is, g = (*g*_1_, *g*_2_,…, $g_{H-1}$) is now shared across loci. This model assumes that the loci, despite their difference in local *N*_e_, respond to the underlying demographic process in the same manner. The rationale comes from the observation that the effects of selection at linked sites (e.g., recurrent selective sweeps, background selection, or the joint effects of the two) can be roughly approximated by a function of the form $N_{e}(t)$ = b(Λ)N(t) (Kim and Stephan 2000; Charlesworth 2012a; Coop and Ralph 2012; Nicolaisen and Desai 2013; Zeng 2013; Corbett-Detig et al. 2015; Zeng and Corcoran 2015). Here, *N*(*t*) is the population size at time *t* in the absence of selection at linked sites. Λ represents parameters of the model under consideration and typically includes the strength of selection, the rate at which selected variants arise, and the recombination rate. The function b(Λ) has relatively weak dependence on the population size. For instance, under background selection, b(Λ) is approximately independent of the population size and is a function of the deleterious mutation rate, the distribution of fitness effects of new deleterious variants, and the recombination rate (Charlesworth 2012a; Nicolaisen and Desai 2013; Zeng 2013; Zeng and Corcoran 2015). Although modeling the effects of selection at linked sites as a reduction in local *N*_e_ is known to be an oversimplification, this approach has been employed by several widely used inference methods (Beaumont 1999; Storz and Beaumont 2002; Hey and Nielsen 2004; Sousa et al. 2013) and should represent a step toward solving the problems caused by ignoring selection at linked sites (Ewing and Jensen 2016; Schrider et al. 2016).

### Dealing with Polarization Errors

So far we have assumed that the uSFS is known. In reality, obtaining the uSFS requires the inference of the ancestral state at polymorphic sites, which can be error-prone (e.g., when sequence divergence to outgroup species is high). It is also known that polarization errors can bias inferences based on the uSFS (Hernandez et al. 2007; Barton and Zeng 2018; Keightley and Jackson 2018). We provide two solutions to this problem. The first is to use the folded SFS (fSFS). Let $D_{k,i}$ be the observed number of segregating sites at which the less frequent allele (minor allele) is represented *i* times (1≤i≤⌊n/2⌋, where ⌊x⌋ is the largest integer that is not greater than *x*). The fSFS for locus *k* is $D_{k}$ = ($D_{k,1},\;D_{k,2}$,…, $D_{k,\left\lfloor n/2\right\rfloor }$), and the overall polymorphism data are D = ($D_{1},\;D_{2}$,…, $D_{K}$). As in the unfolded case, the likelihood of the data can be calculated using the Poisson random field model (see Materials and Methods).

An alternative approach is to explicitly consider polarization error in the model (Williamson et al. 2005; Glémin et al. 2015; Barton and Zeng 2018). When the ancestral state of a segregating site of derived allele frequency *i* is misinferred, it will be incorrectly assigned as a segregating site of derived allele frequency *n* – *i* (0<i<n). Let ϵ_*k*_ be the probability that the ancestral state of a polymorphic site at locus *k* is misinferred. After polarization, the expected number of segregating sites of derived allele frequency *i* is

(1) $$\psi_{k,i}^{*}=(1-\epsilon_{k})\psi_{k,i}+\epsilon_{k}\psi_{k,n-i},$$

where $\psi_{k,i}$ is (true) expected number segregating sites of derived allele frequency *i* and is a function of *θ_k_*, *f_k_*, $g_{k}$, and τ (see eq. 2). As an example, when the above is used with the general model (no divergence), the free parameters include Θ and ϵ_*k*_ (1≤k≤K), which can be estimated by maximum likelihood.

## Results and Discussion

### Properties of the General Model

We evaluated the performance of the general model using X-autosome comparisons as an example. To this end, we employed a two-locus setup and treated locus 1 as the X chromosome (the reference locus) and locus 2 as the autosomes. We generated data from two different models, referred to as Model 1 and Model 2 (table 1). First, let us consider Model 1. It includes several factors that are known to be important for human evolution: changes in the X-autosome ratio of *N*_e_, recent population expansion, and difference in the mutation rate between the X chromosome and autosomes. Here, the simulations were carried out using a demographic model with *H *=* *2 epochs. The *N*_e_ for the X chromosome and the autosomes in epoch 2 (i.e., the most distant epoch) are denoted by *N*_X_ and *N*_A_, respectively. Let $r_{2}=N_{X}/N_{A}$ be the X-autosome ratio of *N*_e_ in epoch 2. At time $\tau_{1}$ before the present, measured in units of $2N_{X}$ generations, the population sizes of the X chromosome and the autosomes changed instantly to $g_{X,1}N_{X}$ and $g_{A,1}N_{A}$, respectively (see supplementary fig. S1, Supplementary Material online, for a graphical representation). As a result, the X-autosome ratio of *N*_e_ in epoch 1 (i.e., the current epoch) is given by $r_{1}=g_{X,1}r_{2}/g_{A,1}$. We assumed that $r_{1}=0.65$ and $r_{2}=3/4$, close to the values reported by Keinan et al. (2009). The shift in the X-autosome ratio of *N*_e_ is accompanied by population expansion characterized by $\tau_{1}=0.1$ and $g_{X,1}=10$. Let *u_X_* and *u*_A_ be the mutation rate per site per generation on the X chromosome and the autosomes, respectively. The corresponding scaled mutation rates are defined as $\theta_{X}=4N_{X}u_{X}$ and $\theta_{A}=4N_{X}u_{A}$ (recall that scaled parameters are defined with respect to the reference locus). We used $\theta_{X}=5.25\times 10^{-4}$ and $\theta_{A}=7.5\times 10^{-4}$. These values give an average autosomal diversity level of 0.001 per site and also reflect the fact that the X chromosome probably have a 30% lower mutation rate than the autosomes (Hodgkinson and Eyre-Walker 2011).

**Table 1.** Mean (standard deviation; SD) of the MLEs for the Parameters of Two Different Two-Locus Models.

	*θ*_X_	*θ*_A_	*r*_1_	*r*_2_	$g_{X,1}$	$\tau_{1}$
Model 1 (true)	$5.25\times 10^{-4}$	$7.5\times 10^{-4}$	0.65	0.75	10	0.1
Mean (SD)	$5.259\times 10^{-4}$ ($5.8\times 10^{-6}$)	$7.53\times 10^{-4}$ ($2.6\times 10^{-5}$)	0.653 (0.05)	0.752 (0.03)	10.0 (0.3)	0.10 (0.003)
Model 2 (true)	$5.25\times 10^{-4}$	$7.5\times 10^{-4}$	0.9	0.75	0.2	0.05
Mean (SD)	$5.261\times 10^{-4}$ ($1.0\times 10^{-5}$)	$7.41\times 10^{-4}$ ($1.0\times 10^{-4}$)	0.89 (0.08)	0.742 (0.10)	0.20 (0.01)	0.051 (0.005)

Note.—Definition of the symbols can be found in supplementary table S1, Supplementary Material online. The population size increases in Model 1, but reduces in Model 2. In both models, the X-autosome ratio of *N*_e_ are different before and after the population size change (as measured by *r*_1_ and *r*_2_). The results are based on 100 simulation replicates. The sample size is 100. Both loci contain 5-Mb sites. The mean number of X-linked and autosomal polymorphic sites are 23,187 and 40,734 under Model 1, and 8,040 and 15,296 under Model 2.

Model 2 is similar to Model 1, except for the following: 1) $r_{1}=0.9$ and $r_{2}=3/4$; 2) the shift in the X-autosome ratio of *N*_e_ coincides with a population size reduction characterized by $\tau_{1}=0.05$ and $g_{X,1}=0.2$. We used Model 2 to assess how the general model fared when the X-autosome ratio of *N*_e_ increased whereas the population size reduced.

We were able to accurately recover all parameters by analyzing only polymorphism data (table 1). Parameter estimation is more difficult under Model 2, as indicated by the higher standard deviation values. This is expected because the population size contraction means that samples generated under Model 2 contain fewer polymorphic sites (see table 1 legend).

Likelihood ratio tests can be readily constructed to ask specific questions of interest. Here, we focus on the following: Test 1—is the mutation rate different between the X chromosome and the autosomes (a model with $\theta_{X}=\theta_{A}$ vs. the full model; degree of freedom [df] = 1)? Test 2—is there evidence for the X-autosome ratio of *N*_e_ being significantly different from 0.75 (a model with $r_{1}=r_{2}=3/4$ vs. the full model; df = 2)? Test 3—has the X-autosome ratio of *N*_e_ changed between epochs (a model with $r_{1}=r_{2}$ vs. the full model; df = 1)? These tests were applied to the simulated data used in table 1, and the results are shown in table 2. Test 1 is less powerful under Model 2 than under Model 1, which is an expected consequence of a drop in the number of polymorphic sites. In contrast, Test 2 has higher power under Model 2 than under Model 1, and the power of Test 3 is comparable between the models. These observations indicate that the number of polymorphic sites is not the only factor that affects statistical power.

**Table 2.** Power (%) of the Three Likelihood Ratio Tests.

Model	Test 1	Test 2	Test 3
Model 1	100	84	83
Model 2	67	98	78

Note.—Model 1 and Model 2 are the same as those used in table 1; so are the number of replicates, sample size, and locus length. Each sample was analyzed using the likelihood ratio tests described in the main text. The values above are the frequency at which the null model is rejected at a 5% significance level.

Overall, the simulations suggest that polymorphism data can be used to obtain information about X-autosome differences in *N*_e_ and/or *u*. The power of these analyses depends in a complex way on both the sample size and the demographic history. It should also be pointed out that the divergence-based version of the general model is not suitable for analyzing data sets containing only two loci. This is because the data contain very little information about the parameters *c* and *t*. In fact, simulations suggest that, when this is the case, including divergence data can lead to biases in parameter estimation (supplementary table S2, Supplementary Material online).

### Properties of the Simplified Model

This model is suitable for analyzing data collected from multiple autosomal or sex-linked loci. We will start by analyzing data sets consisting of a small number of loci, in order to demonstrate several important properties of the model. We will then consider data sets with many loci, which represents a much more challenging problem.

### Data Sets with a Small Number of Loci

**Fig. 1.**
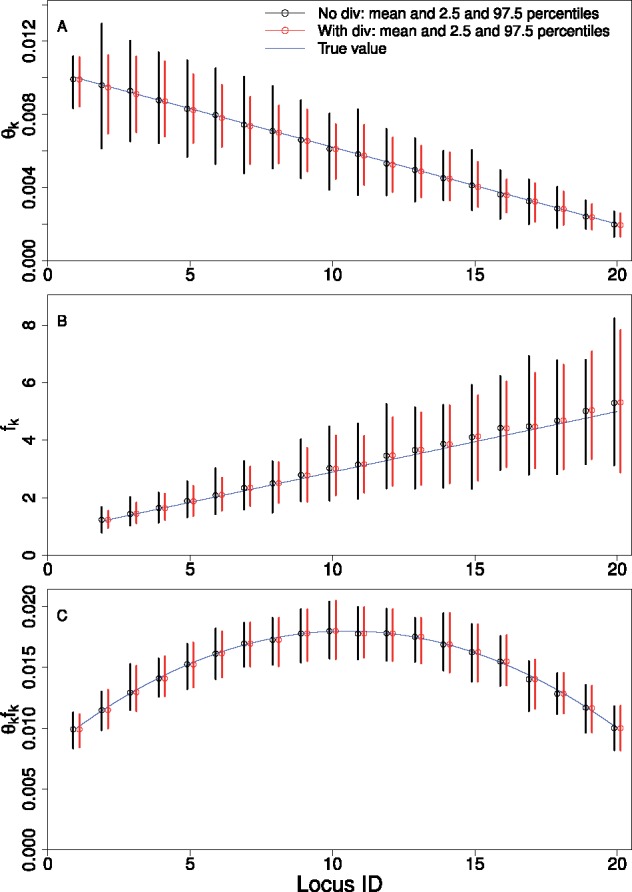
MLEs obtained by fitting the simplified model to simulated data from 20 loci. Each locus is 5 kb long. The solid blue line in each plot shows the true parameter values across loci. The population size expanded recently with parameters $g_{1}=10$ and $\tau_{1}=0.5$. The results are based on 100 replicates. The sample size is 100.

We analyzed 100 simulated data sets. Each data set contains the uSFS from 20 loci, and the sample size is 100. All loci are 5 kb long. The scaled parameters are defined with respect to *N*_1_, the *N*_e_ at locus 1 in the most distant epoch (i.e., locus 1 is the reference locus). The scaled mutation rate *θ_k_* (1≤k≤20) vary linearly across loci, with $\theta_{1}/\theta_{20}=5$ (blue line in fig. 1*A*). The *f_k_* (1≤k≤20) also vary linearly with $f_{20}/f_{1}=5$ (blue line in fig. 1*B*). The demographic model has *H *=* *2 epochs. At time $\tau_{1}=0.5$ before the present, the population size increased 10-fold (i.e., $g_{1}=10$). To model divergence, we assumed that the population of the ancestral species was larger with *c *=* *2. The scaled divergence time is *t *=* *8. With these parameter values, the expected divergence level at locus 1 is 0.1 per site.

The simulated data were first analyzed by combining the uSFS from the 20 loci into a single uSFS (i.e., disregarding variation in *N*_e_ and *u*). Estimates of *g*_1_ and $\tau_{1}$ were obtained by fitting the combined data to a demographic model with a one-step change in population size. The mean and the interval between the 2.5 and 97.5 percentiles are 9.20 and [8.62, 9.63] for *g*_1_, and 0.58 and [0.54, 0.61] for $\tau_{1}$. Both estimates are biased, and neither of the intervals overlaps the true value. Thus, ignoring heterogeneity in *N*_e_ and *u* can lead to high statistical support for biased estimates.

The simulated data were then analyzed by the simplified model, both with and without using the divergence data. From table 3, we can see that the model can provide unbiased estimates for both *g*_1_ and $\tau_{1}$, regardless of whether divergence data were used. The standard deviation (SD) values in table 3 suggest that estimates of $\tau_{1}$ are somewhat less variable with divergence data. The model is also able to provide accurate estimates of *c* and *t*, in contrast to the two-locus case (supplementary table S2, Supplementary Material online).

**Table 3.** Parameter Estimates Obtained by Applying the Simplified Model to Simulated Data Sets Containing Either 20 or 500 Loci.

Data	Mean (SD)			
	*g*_1_	$\tau_{1}$	*c*	*t*
True (20 loci)	10	0.5	2	8
With div	10.1 (0.26)	0.51 (0.06)	2.0 (0.2)	8.1 (0.9)
No div	10.1 (0.26)	0.51 (0.08)	—	—
True (500 loci)	10	0.5	2	6
With div	10.0 (0.04)	0.50 (0.04)	2.0 (0.04)	6.0 (0.5)
No div	10.0 (0.04)	0.50 (0.05)	—	—

Note.—The cases with 20 loci are the same as those presented in figure 1. The locus length is 5 kb, and the results are based on 100 replicates and a sample size of 100. For the cases with 500 loci, *θ_k_* and *f_k_* were sampled from the gamma distributions described in the main text. The locus length is 10 kb, and the results are based on 50 replicates and a sample size of 50. The demographic model is the same in all cases, and is characterized by *g*_1_ and $\tau_{1}$.

Regarding *θ_k_* and *f_k_*, the estimates are also unbiased (fig. 1). The addition of divergence data appears to slightly lower the variance of the estimates. In figure 1*B*, we can see that the variance of the *f_k_* estimates tends to be larger for loci with a higher index, whereas the variance of the estimates of the composite parameter $\theta_{k}f_{k}$ is more uniform across loci (fig. 1*C*). To see why, we first note that $\theta_{k}f_{k}$ is the total scaled mutation rate at locus *k* in the most distant epoch (i.e., scaled by the *N*_e_ at locus *k* instead of the *N*_e_ at the reference locus). The information the model uses to separate *θ_k_* and *f_k_* comes in part from the distortion of the local genealogy caused by the recent expansion. For locus *k*, the rate of coalescence (in units of $2N_{1}$ generations) between the present and the time of expansion is $1/(f_{k}g_{1})$. Thus, coalescence occurs at a slower rate at loci with a larger local *N*_e_ (i.e., a higher true value of *f_k_* in fig. 1*B*). In the most extreme scenario when *f_k_* is so large that $1/(f_{k}g_{1})$ approaches zero, the local genealogy will be indistinguishable from that expected under the equilibrium model. In this case, the likelihood surface will contain a ridge on which both *θ_k_* and *f_k_* vary with the product $\theta_{k}f_{k}$ held constant, making it impossible to separate *θ_k_* and *f_k_*. As *f_k_* is large when *k* is large (fig. 1*B*), the increase in variance reflects the increase in difficulty in separating *θ_k_* and *f_k_*. This suggests that the ability to estimate *θ_k_* and *f_k_* separately at locus *k* depends on both the demographic history and properties specific to the locus itself.

Finally, we repeated the above analyses, but used locus 20 as the reference locus instead. This has little effect on the results. For instance, the mean (SD) of the MLEs of *g*_1_ is 10.1 (0.29) without divergence data, and 10.1 (0.29) with divergence data (cf. table 3). As in figure 1, estimates of *f_k_* are more variable for loci with a higher local *N*_e_ (supplementary fig. S2, Supplementary Material online). Thus, the choice of reference locus may be relatively unimportant.

### Data Sets with a Large Number of Loci

We analyzed 50 simulated data sets. Each data set contains uSFS from 500 loci, and the sample size is 50. The loci are 10 kb in length. As above, the scaled parameters are defined with respect to *N*_1_, the *N*_e_ at locus 1 in the most distant epoch. In each replicate, we sampled *θ_k_* from a gamma distribution with shape $a_{\theta }$ = 3 and scale $b_{\theta }=0.005$, and *f_k_* from a gamma distribution with shape $a_{f}=5$ and scale $b_{f}=0.2$. For divergence, we used *c *=* *2 and *t *=* *6 in all replicates. The average diversity and divergence levels under these parameters are 1.5% and 12%, respectively, which are close to those observed at putatively neutral sites in short introns on the autosomes of *D. melanogaster* (using *D. simulans* as an outgroup; Jackson et al. 2015). The demographic model is the same as that used in figure 1. The use of the gamma distribution was inspired by a previous study (Gossmann et al. 2011), but the values of the shape and scale parameters are somewhat arbitrary. Our treatment also does not consider distortions in the shape of the SFS caused by selection at linked sites. These simplifications were made on consideration of the complexity of the inference problem, so that we could assess the model’s performance in a relatively simple setting.

The data shown in table 3 suggest that *g*_1_, $\tau_{1}$, *c*, and *t* can all be estimated accurately. As a different set of *θ_k_* and *f_k_* were sampled from the gamma distributions in each replicate, we assessed the accuracy of the model by calculating the slope and intercept of the linear regression of the MLEs of *θ_k_* and *f_k_* over their true values. For *θ_k_*, the mean (SD) for the slopes and intercepts are 1.00 (0.09) and $6.6\times 10^{-5}$ ($5.0\times 10^{-5}$) with divergence data, and 0.99 (0.10) and $1.7\times 10^{-4}$ ($8.7\times 10^{-5}$) without divergence data. For *f_k_*, these are 0.95 (0.08) and 0.05 (0.01) with divergence data, and 0.93 (0.10) and 0.07 (0.01) without divergence data. Thus, as above, the inclusion of divergence data seems to increase accuracy and lower variance. Compared with *f_k_*, the regression lines for *θ_k_* have slopes closer to 1 and intercepts closer to 0, suggesting that *θ_k_* tends to be more accurately estimated using this method.

As discussed in the previous section, when the data do not contain enough information, *θ_k_* and *f_k_* tend to form a ridge in the likelihood surface. This can create an artificial negative correlation between these two parameters, which may be problematic if the MLEs of *θ_k_* and *f_k_* are to be used for detecting association with other genomic variables (e.g., GC content, recombination rate). As the true values of *θ_k_* and *f_k_* were sampled from two independent probability distributions in the simulations, their MLEs should be uncorrelated. However, when making inferences on polymorphism data alone, the MLEs of *θ_k_* and *f_k_* are significantly negatively correlated in 16% of the simulation replicates (based on Kendall’s *τ* and a significance level of 5%). In contrast, for estimates based on both polymorphism and divergence, only 2% of the replicates show a significant negative correlation, suggesting that the addition of divergence data has increased the model’s ability to separate variation in *N*_e_ from that in *u*. It should be pointed out that this requires the divergence level to be sufficiently large. For instance, if we keep all parameters the same as above, but reduce *t*, the scaled divergence time, such that the expected divergence level drops from 12% to 6%, the MLEs of *θ_k_* and *f_k_* are significantly correlated in 8% of the replicates. In practice, the “required” level of divergence is a function of the demographic history of the ingroup species, lengths of the loci, and the number of alleles in the sample.

### Implications of the Results Based on the Simplified Model

The results presented above suggest that disregarding variability in *N*_e_ and *u* can lead to biased demographic inferences. The new methods can solve this problem and help to quantify this heterogeneity across loci. It is, however, important to note that the ability to separate *θ_k_* and *f_k_* depends on several factors—the demographic history, the local effective population size, and the sample size (in terms of both the number of alleles and locus lengths). When there is insufficient information, the ridge along $\theta_{k}f_{k}$ tends to create a negative correlation between the MLEs of *θ_k_* and *f_k_*. There is some evidence that the inclusion of divergence data can help to counter this tendency, and (moderately) lower variance in parameter estimation (fig. 1 and table 3). It should, however, be noted that we have used a highly simplifying model of divergence. It is of interest to incorporate complications such as nonequilibrium substitution patterns in the future by using, for instance, the framework of Matsumoto et al. (2015).

The above discussion is relevant to other methods that allow *N*_e_ and *u* to vary across loci, especially when considering that these methods do not use divergence data to help the inference (Beaumont 1999; Storz and Beaumont 2002; Hey and Nielsen 2004; Sousa et al. 2013). Thus, the simulations highlight a major challenge in population genetic data analysis—although many important questions in evolution can be studied by detecting differences in *N*_e_ and *u*, the fact that diversity patterns are determined by the composite parameter $N_{e}u$ means that separating these two parameters is inher—ently difficult. The same applies to the analysis of data collected from subdivided populations. Here the composite parameter $N_{e}m$, where *m* is the migration rate, is inversely correlated to the level of differentiation between populations. As a result, distinguishing between the following two causes of locally elevated levels of differentiation may not be straightforward (Cruickshank and Hahn 2014): 1) Loci have smaller *m* due to their involvement in selection against gene flow (Wolf and Ellegren 2017) and 2) loci have reduced *N*_e_, but not *m*, as a result of background selection (Zeng and Corcoran 2015). Therefore, how to further increase the statistical power and robustness of the methods cited above warrants further investigation.

### Application to the *D. simulans* Data

#### X-autosome Comparisons Based on the General Model

Our data set contains 21 alleles collected from the putative ancestral range in Madagascar (Jackson et al. 2017; see table 1 therein for values of summary statistics such as the nucleotide diversity [*π*] and Tajima’s *D*). To avoid complication caused by selection on synonymous codon usage, we considered sequence variability on putatively neutrally evolving sites in short introns (i.e., positions 8–30 bp of introns <66 bp; see also Parsch et al. 2010).

Comparing the X chromosome and the autosomes (A), the diversity ratio is $\pi_{X}/\pi_{A}=0.0195/0.0311=0.63$. This is lower than the “null” value of 0.75 expected when there is a 1:1 sex ratio and no difference in reproduction success between sexes (Charlesworth 2009). However, the population is not at equilibrium, as suggested by the negative Tajima’s *D* value of −1.46 on the X chromosome and −1.19 on the autosomes. It is known that changes in population size can perturb $\pi_{X}/\pi_{A}$ away from 0.75 (Pool and Nielsen 2007). Thus, the observed $\pi_{X}/\pi_{A}$ ratio can potentially be explained by a combination of the following factors: 1) recent demography; 2) difference in *N*_e_ between X and A as a result of an unequal sex ratio, difference in the mode of inheritance, and/or variation in reproductive success between sexes; and 3) difference in the mutation rate between X and A.

To determine which of the three factors may have had an effect on $\pi_{X}/\pi_{A}$, we fitted the general model to the uSFS, with the ancestral state at polymorphic sites inferred using *D. melanogaster* as an outgroup and maximum parsimony. A model with *H *=* *2 epochs fits the data well, except for the uptick toward the high-frequency end of the uSFS (table 4 and supplementary fig. S3, Supplementary Material online). Increasing the number of epochs to *H *=* *3 does not significantly improve the fit, suggesting that the uptick is most probably a result of polarization error (supplementary fig. S3, Supplementary Material online). As the sample size is relatively small, using the fSFS is likely to lead to a significant loss of power. Thus, we adopted the alternative approach and introduced two new parameters, ϵ_X_ and ϵ_A_, for modeling polarization errors in the X-linked and autosomal data set, respectively. This model explains the observed uSFS significantly better than the no-error model (supplementary fig. S4, Supplementary Material online). This is further confirmed by the fact that the 95% CIs for the two polarization error parameters have lower bounds >0 (table 4). Adding another epoch to the model does not significantly increase the goodness of fit ($P_{b}=0.51$, where the subscript *b* signifies that the *P*-value was obtained by bootstrapping). Thus, we refer to the model with *H *=* *2 and polarization error as the best-fit model, and use it in the subsequent analyses.

**Table 4.** Parameter Estimates Obtained by Fitting Two Models to the uSFS from *Drosophila simulans*.

Model	MLE and 95% CI of Parameters						
*H *=* *2	*θ*_X_	*θ*_A_	*r*_1_	*r*_2_	$g_{X,1}$	$\tau_{1}$		
No pol err	0.015	0.024	1.99	1.00	11.88	0.40		
	[0.013, 0.016]	[0.019, 0.028]	[1.26, 2.80]	[0.78, 1.18]	[9.35, 14.93]	[0.33, 0.49]		
*H* = 2	*θ*_X_	*θ*_A_	*r*_1_	*r*_2_	$g_{X,1}$	$\tau_{1}$	ɛ_X_	ɛ_A_
With pol err	0.011	0.019	1.91	1.03	12.60	0.67	0.06	0.05
	[0.010, 0.013]	[0.014, 0.025]	[1.24, 2.63]	[0.77, 1.33]	[9.99, 15.43]	[0.51, 0.86]	[0.05, 0.07]	[0.05, 0.06]

Note.—Both models have *H *=* *2 epochs. The second model contains two extra parameters, ɛ_X_ and ɛ_A_, for modeling polarization errors in the X-linked and autosomal data set, respectively. The 95% CIs were obtained by analyzing 100 bootstrap samples. The bootstrap samples were generated by sampling the short introns with replacement, while keeping the numbers of X-linked and autosomal introns the same as in the real data set.

The MLEs of the parameters in the best-fit model are presented in table 4. Consistent with the negative Tajima’s *D* values, $g_{X,1}$ is significantly >1, providing support for a recent population expansion ($P_{b}<0.01$). The X chromosome mutates at a lower rate than the autosomes, and the MLE of $\theta_{X}/\theta_{A}$ is 0.59 (95% CI = [0.49, 0.68]), which is significantly smaller than 1 ($P_{b}<0.01$). The MLE of *r*_1_, the X-autosome ratio of *N*_e_ in the current epoch (i.e., epoch 1), is 1.91, and that of *r*_2_, the *N*_e_ ratio in epoch 2 (i.e., before the expansion), is 1.03. Bootstrapping suggests that both *r*_1_ and *r*_2_ are significantly higher than 0.75 ($P_{b}<0.01$), and that the *N*_e_ ratio is probably different between the two epochs ($P_{b}<0.01$). Thus, all the three factors listed above may have affected the observed $\pi_{X}/\pi_{A}$.

#### Implications of the Results Obtained from the *D. simulans* Data

The fact that the MLE of the X-autosome mutation rate ratio is 0.59 is interesting and lends support to the existence of male-driven evolution in *Drosophila* (Bachtrog 2008). However, our estimate is significantly smaller than the X-autosome divergence rate ratio of 0.91 estimated on the same set of short introns by Charlesworth et al. (2018). The reason for this difference is unclear. It is possible that the mutation rate has evolved. The fact that substitution patterns are significantly different between the *D. simulans* and *D. melanogaster* lineages is potentially consistent with this, although other explanations have been put forward (Jackson et al. 2017). Alternatively, the difference may be caused by the fact that the general model does not consider the potential existence of a GC-favoring force acting on short introns, possibly due to GC-biased gene conversion (Jackson et al. 2017). However, the MLE of the X-autosome mutation rate ratio is still 0.59 when applying the model to variants that are unaffected by GC-biased gene conversion (i.e., a reduced data set containing polymorphic sites between A and T, and those between G and C). Thus, what causes the difference requires further investigation. Nonetheless, both our analysis and the analysis of Charlesworth et al. (2018) suggest that the X chromosome has a lower mutation rate than the autosomes, which may have direct bearing on the study of the faster-X hypothesis in *Drosophila* (Charlesworth et al. 2018).

The MLE of *r*_2_ (the long-term X-autosome ratio of *N*_e_ before the expansion) is 1.03. It is close to the upper limit of 9/8, expected when there is an extremely female-biased sex ratio or substantially higher variance in reproductive success in males (Charlesworth 2009; Webster and Wilson Sayres 2016). The proximity to the upper limit could be a result of statistical noise, as suggested by the wide 95% confidence interval (table 4). Nevertheless, the fact that *r*_2_ is significantly higher than 0.75 lends support to the possibility of a female-biased sex ratio or high variance in male reproductive success. Further studies should investigate whether *r*_2_ may have also been influenced by other factors such as mate pairing practices, selection at linked sites, and sex-biased demographic changes (Charlesworth 2001, 2012b; Keinan et al. 2009; Evans and Charlesworth 2013; Webster and Wilson Sayres 2016).

The MLE of *r*_1_ (the X-autosome ratio of *N*_e_ in the most recent epoch) is 1.91, significantly higher than the upper limit of 9/8 (Charlesworth 2009; Webster and Wilson Sayres 2016). However, the simulation results presented in supplementary table S3, Supplementary Material online, suggest that the estimation of *r*_1_ may be liable to upward biases when there are very recent events that are difficult for a sample of 21 alleles to detect. The main reason is that the smaller number of polymorphic sites in the X-linked data set (due to its lower mutation rate and shorter length) restricts its ability to detect recent events. Thus, further research using a much larger sample is needed to rule out methodological artifacts as the reason for the high estimate of *r*_1_. Fortunately, this potential power issue does not affect the estimation of the $\theta_{X}/\theta_{A}$ ratio and *r*_2_. Thus, the conclusions of a lower mutation rate on the X chromosome and a potentially female-biased sex ratio should be robust.

## Conclusion

In this study, we show that it is possible to use polymorphism data to jointly infer past changes in population size and variation in *N*_e_ and *u*, provided that the population is not at equilibrium. These methods are capable of handling a large number of loci and many alleles (thousands). Including divergence data can increase the statistical power in some cases. However, because the mutation pattern itself may evolve (Smith et al. 2018), care should be exercised when choosing the outgroup. We have assumed that the population size changes in a stepwise manner, but this assumption can be readily relaxed (Polanski and Kimmel 2003; Bhaskar et al. 2015; Gao and Keinan 2016). It is important to note that *N*_e_ and *u* are confounded (similarly, *N*_e_ and *m*, the migration rate, are confounded; Sousa et al. 2013). This makes separating these parameters inherently difficult. This difficulty can in part be dealt with by increasing the sample size (both the locus length and the number of alleles), which has become feasible, thanks to advances in sequencing technologies. Our analyses have shown that the modeling framework developed herein provides an effective way of analyzing the data and can be used to study a variety of questions in different organisms.

## Materials and Methods

### Further Details of the Models

Assuming neutrality and an infinite-sites model of mutation, the expected number of segregating sites of derived allele frequency *i* in a sample of *n* alleles taken from locus *k* is given by

(2) $$\psi_{k,i}=\psi_{k,i}(\theta_{k},f_{k},g_{k},\tau )=m_{k}\theta_{k}\varphi_{k,i}(f_{k},g_{k},\tau ),$$

where 1≤i<n, *m_k_* is the length (in basepairs) of locus *k*, $2\varphi_{k,i}$ is the expected total length of branches in the coalescent genealogy that have *i* descendants in the sample (Wakeley 2009, Section 4.1.3). Marth et al. (2004) derived an explicit expression of $\varphi_{k,i}$, which we have rearranged in the following form:

(3) $$\varphi_{k,i}=\varphi_{k,i}(f_{k},g_{k},\tau )=f_{k}[\frac{g_{k,1}}{i}+A_{k,i}(f_{k},g_{k},\tau )],$$

where

(4) $$A_{k,i}(f_{k},g_{k},\tau )$$

$$=\sum_{h=1}^{H-1}\left(g_{k,h+1}-g_{k,h}\right)\sum_{j=2}^{n}e^{-\left(\begin{array}{c} j\\ 2 \end{array}\right)\sum_{l=1}^{h}\frac{\tau_{l}}{f_{k}g_{k,l}}}B_{i}(j),$$

(5) $$B_{i}(j)=\frac{1}{i}{\left(\begin{array}{c} n-1\\ i \end{array}\right)}^{-1}\sum_{b=2}^{j}\left(\begin{array}{c} n-b\\ i-1 \end{array}\right)C(b,j),$$

(6) $$C(b,j)=\prod_{l:l\neq j;b\leq l\leq n}\frac{l(l-1)}{l(l-1)-j(j-1)},$$

and $g_{k,H}=1$.

We use the Poisson random field model, which assumes that the sites are unlinked, to calculate the (composite) likelihood of the uSFS (Sawyer and Hartl 1992; Bustamante et al. 2001). Specifically, the probability that we observe $d_{k,i}$ segregating sites of derived allele frequency *i* at locus *k* is given by $e^{-\psi_{k,i}}{\left(\psi_{k,i}\right)}^{d_{k,i}}/(d_{k,i}!)$. The log likelihood of the data is

(7) $$\begin{array}{c} L(\Theta |d)=\text{ln}[\prod_{k=1}^{K}\prod_{i=1}^{n-1}e^{-\psi_{k,i}}\frac{{\left(\psi_{k,i}\right)}^{d_{k,i}}}{d_{k,i}!}]\\ =\sum_{k=1}^{K}\sum_{i=1}^{n-1}[-\psi_{k,i}+d_{k,i}\text{ln}\psi_{k,i}]+C \end{array},$$

where *C* is a constant that depends only on the data, and is therefore omitted from the calculation.

An alternative way of calculating the likelihood of the uSFS is to condition on the segregating sites (Williamson et al. 2005). To this end, we note that the probability that a particular SNP is of derived allele frequency *i* is given by $\zeta_{k,i}=\psi_{k,i}/\psi_{k}=\varphi_{k,i}/\varphi_{k}$, where $\psi_{k}=\sum_{j=1}^{n-1}\psi_{k,j}$ and $\varphi_{k}=\sum_{j=1}^{n-1}\varphi_{k,j}$. Importantly, $\zeta_{k,i}$ is independent of the mutation rate. Therefore, assuming that the sites are unlinked, the log likelihood is

(8) $$\begin{array}{c} L(\Theta^{*}|d)=\text{ln}{\left[\prod_{k=1}^{K}\prod_{i=1}^{n-1}(\zeta_{k,i}\right)}^{d_{k,i}}]\\ =\sum_{k=1}^{K}\sum_{i=1}^{n-1}d_{k,i}[\text{ln}\varphi_{k,i}-\text{ln}\varphi_{k}] \end{array},$$

where $\Theta^{*}$ represents all the parameters in Θ less *θ_k_* (1≤k≤K). This equation is equivalent to the profile likelihood function described by Bhaskar et al. (2015) and is computationally more efficient than equation (7) by reducing the dimensionality of the problem. Once MLEs of *f_k_*, $g_{k}$, and τ have been found, we can use them to calculate $\varphi_{k}$ and estimate *θ_k_* as $S_{k}/(m_{k}\varphi_{k})$, where $S_{k}=\sum_{i=1}^{n-1}d_{k,i}$ is the total number of segregating sites from locus *k* (Bustamante et al. 2001; Bhaskar et al. 2015).

To include divergence data, we assume that the number of substitutions follows a Poisson distribution with mean *λ_k_*. The augmented version of equation (7) can be written as

(9) $$\begin{array}{c} L(\Theta ,c,t|d,X)\\ =\sum_{k=1}^{K}[-\lambda_{k}+x_{k}\text{ln}\lambda_{k}+\sum_{i=1}^{n-1}(-\psi_{k,i}+d_{k,i}\text{ln}\psi_{k,i})], \end{array}$$

where constants dependent on the data are omitted, and X = (*x*_1_, *x*_2_,…, *x_K_*) are the observed number of substitutions. It should be noted that the information about *c* and *t* comes from the variation in divergence level between loci. Thus, although the composite parameter $cf_{k}+t$ should be estimated accurately, the model may have difficulty teasing *c* and *t* apart when there is only a small number of loci and/or when $cf_{k}\ll t$ (for 1≤k≤K).

To calculate likelihood of the fSFS, we define $\Psi_{k,i}=\psi_{k,i}+\delta (i<n-i)\psi_{k,n-i}$, where δ(y)=1 if the condition *y* is true and 0 otherwise. Likelihood functions corresponding to equations (7–9) can be obtained by changing the upper limit of the second summation from n−1 to ⌊n/2⌋ and replacing $d_{k,i}$ by $D_{k,i}$, and $\psi_{k,i}$ by $\Psi_{k,i}$.

Finally, to explicitly consider polarization errors, we introduce parameters ϵ_*k*_ into the model (1≤k≤K). The likelihood functions are analogous to equations (7–9), but with $\psi_{k,i}^{*}$ (see eq. 1) in place of $\psi_{k,i}$. Note that the uSFS must be used in this case, as the fSFS contains no information about polarization error rates.

### Computational Details

Calculation of $\psi_{k,i}$ (see eq. 2) is complicated by the presence of the alternating terms *C*(*b*, *j*) (see eq. 6), which can result in catastrophic cancellation during standard double-precision floating-point computations. Marth et al. (2004) dealt with this problem by using numerical libraries that performed arbitrary precision arithmetic. However, these libraries tend to be slow, especially when the sample size is large. For instance, a sample of 1,000 requires a numerical precision of ∼500 decimal places, which is orders of magnitude slower than the standard double-precision arithmetic (16-digit precision). Here, we observe that, in our new representation of the result of Marth et al. (2004) (see eq. 3–6), $B_{i}(j)=\frac{1}{j(j-1)}W_{i,j}^{n}$, where $W_{i,j}^{n}$ is given by equation (10) in Polanski and Kimmel (2003). Thus, we can obtain $W_{i,j}^{n}$ (and then $B_{i}(j)$) using the recursion equations derived by Polanski and Kimmel (2003, see eqs. 13–15 therein). These equations can be evaluated using the standard double-precision arithmetic and are known to be numerically stable and very fast.

Due to the introduction of locus-specific parameters, evaluating the likelihood function requires the calculation of *K* locus-specific expected SFSs. As the order in which the expected SFSs are obtained is unimportant, the computation can be sped up by distributing the work across multiple CPU cores. This is achieved here via OpenMP (http://www.openmp.org/).

### Analysis of the Simulated Data

We performed parameter estimation using our program, varne, on random samples simulated using Mathematica (http://www.wolfram.com/). The computations in Mathematica were carried out using a very high precision level with 315 digits. Because the generation of simulated data was separate from the numerical routines we used in varne, this setup can verify the numerical robustness of varne. Unless stated otherwise, 100 data sets were generated for each parameter combination and only uSFSs were used.

To obtain MLEs of the parameters, we used gradient-based optimization algorithms implemented in the NLopt library (http://ab-initio.mit.edu/nlopt). Partial derivatives were obtained by analytically differentiating equation (2) with respect to the parameters of the model. This is numerically much more stable than the finite difference method. Wherever possible, the profile likelihood (eq. 8) was used in favor of its higher computational efficiency. To ensure that the global maximum was found, the optimization algorithm was run multiple times, each starting from a randomly chosen point in the parameter space. The most complex case has *H *=* *2 epochs and contains both polymorphism and divergence data from 500 loci. The corresponding model has 1,003 parameters. The optimization algorithms seem to cope well with the high dimensionality of the problem—the MLE was typically found by running the algorithm for <50 times.

### Analysis of the *D. simulans* Data

We downloaded raw read data in fastq format for 21 isofemale lines of *D. simulans* collected from Madagascar from the European Nucleotide Archive (study accession numbers: PRJEB7673; PRJNA215932). These samples were previously described by Jackson et al. (2017). We mapped the reads to version 2.02 of the *D. simulans* genome (FlyBase release 2017_04) using BWA MEM (Li 2013), then sorted, merged and marked duplicates on the resulting BAM files using Picard Tools version 2.8.3 (https://broadinstitute.github.io/picard/). We called variants separately for each individual line using the HaplotypeCaller tool from GATK version 3.7 (McKenna et al. 2010), with the options –emitRefConfidence, BP_RESOLUTION and –max-alternate-alleles 2, then made per-chromosome VCF files for the whole population using the GATK v3.7 tools combineGVCFs and genotypeGVCFs. The sequencing depth per sample ranged from 54× to 100×. All the scripts necessary for downloading the fastq files and calling variants are available at https://github.com/benjamincjackson/dsim_variant_pipeline_ref_v2.02.git.

Multispecies alignment was performed between the reference genomes of *D. simulans* (v2.02), *D. melanogaster* (v5.57), and *D. yakuba* (v1.3) using the same MULTI-Z pipeline as described by Barton and Zeng (2018). We used the information in the header lines of the FlyBase fasta file of introns for version 2.02 of the *D. simulans* reference (available from ftp://ftp.flybase.net/genomes/Drosophila_simulans/dsim_r2.02_FB2017_04/fasta/dsim-all-intron-r2.02.fasta.gz) to extract coordinates of the 8–30 bp region of introns that were ≤65 bp in length, after checking that this region did not overlap with an exon, an intron of length more than 65 bp, or the non-8–30 bp portion of an intron of length ≤65 bp, using information from the gff format annotation of the *D. simulans* genome v2.02 (available from ftp://ftp.flybase.net/genomes/Drosophila_simulans/dsim_r2.02_FB2017_04/gff/dsim-all-r2.02.gff.gz).

Using these coordinates we made fasta files containing sequences from the 21 *D. simulans* lines as well as from the *D. melanogaster* and *D. yakuba* reference sequences, keeping only sites that met the following criteria: no more than two alleles in the polymorphism data set; phred-scaled quality score (QUAL) > 30; no missing data in any of the polymorphism or outgroup samples; not soft-masked as being repetitive in the multiple alignment step; no overlap with indels in the variant callset. For positions that still contained residual heterozygosity after the inbreeding process we chose one allele with probability proportional to the read depth for each allele at that site, following Jackson et al. (2017).

We extracted all autosomal (excluding the fourth chromosome) and X-linked polymorphic sites. To unfold the SFS, we used the *D. melanogaster* reference genome as an outgroup and the maximum parsimony principle. When analyzing these data using the general model, we did not require that the X-autosome ratio of *N*_e_ varied between 9/16 and 9/8, with the lower bound corresponding to the case where there is only one breeding female (or much higher variance in reproductive success in females than males), and the upper bound corresponding to the case where there is only one breeding male (or much higher variance in reproductive success in males than females). This choice is different from some previous studies (Clemente et al. 2018) and means that our models do not regard deviation from a 1:1 sex ratio as the only reason why the X-autosome ratio of *N*_e_ departs from the “null” value of 0.75. For example, it is possible for selection at linked sites to reduce diversity more substantially on the autosomes than on the X chromosome (Charlesworth 2012b). If this is combined with a female-biased sex ratio, the X-autosome ratio of *N*_e_ may go above 9/8.

We used bootstrapping to access uncertainties in the parameter estimation. We assumed that all sites within a short intron were completely linked, and different short introns were unlinked. These assumptions should be reasonable because each short intron region is only 23 bp long, and we expect linkage disequilibrium to decay very rapidly in *D. simulans* (True et al. 1996). The results were obtained by analyzing 100 bootstrap samples. The bootstrap samples were generated by sampling the short introns with replacement, while keeping the numbers of X-linked and autosomal introns the same as in the real data set.

### Software Availability

The models presented here have been implemented in a computer package varne, which can be downloaded from http://zeng-lab.group.shef.ac.uk/.

## Supplementary Material

Supplementary data are available at *Molecular Biology and Evolution* online.

## Supplementary Material

Supplementary Data
