# Supplementary material for: Methods for Estimating Demography and Detecting Between-Locus Differences in the Effective Population Size and Mutation Rate
Source: Mol Biol Evol. 2018 Nov 14;36(2):423–33. doi: 10.1093/molbev/msy212 (PMC6409433; doi:10.1093/molbev/msy212)

Table S1: Symbols and definitions. Greek letters appear first, followed by English letters. The letters are ordered alphabetically.

Symbol	Definition
ϵ_k	The polarisation error rate at locus k.
θ_k	$4N_r u_k$, the scaled mutation rate for locus k, where N_r is the N_e at the reference locus.
θ_X and θ_A	$4N_X u_X$ and $4N_X u_A$, the scaled mutation rate for the X chromosome and autosomes, respectively (the X chromosome as the reference).
$\boldsymbol{\Theta}$	$\boldsymbol{\tau}$, θ_k, f_k, $\boldsymbol{g}_k$ ($1 \leq k \leq K$ and $f_1 = 1$).
λ_k	The expected divergence level at locus k.
τ_h	The duration of epoch h, in units of $2N_r$ generations, where N_r is the N_e at the reference locus.
$\boldsymbol{\tau}$	τ_1, τ_2, ..., τ_{H-1}.
$\psi_{k,i}$ (or $\psi^*_{k,i}$)	The expected number of polymorphic sites of derived allele frequency i at locus k without (or with) polarisation error.
c	The relative size of the population of the ancestral species.
$d_{k,i}$ (or $D_{k,i}$)	The number of polymorphic sites at locus k at which the derived (or the minor allele) is represented i times.
$\boldsymbol{d}$ (or $\boldsymbol{D}$)	The unfolded (or folded) SFS from all K loci, respectively.
f_k	The effective population size at locus k in the most distant epoch is $f_k N_r$, where N_r is the N_e at the reference locus.
$g_{k,h}$	The effective population size at locus k in epoch h is $f_k g_{k,h} N_r$, where N_r is the N_e at the reference locus.
$g_{X,h}$ and $g_{A,h}$	The same as $g_{k,h}$, but are for the X chromosome and autosomes, respectively.
$\boldsymbol{g}_k$	$g_{k,1}$, $g_{k,2}$, ..., $g_{k,H-1}$.
$\boldsymbol{g}$	g_1, g_2, ..., g_{H-1}. In the simplified model, $g_h = g_{k,h}$ for all k.
H	The total number of epochs or the index of the most distant epoch. The population size is constant when $H = 1$.
K	The total number of loci.
m_k	The length of locus k in basepairs.
N_1	The effective population size at locus 1 in the most distant epoch.
N_X and N_A	The effective population size of the X chromosome and autosomes in the most distant epoch.
n	The sample size (i.e., the number of alleles in polymorphism dataset).
r_h	The X-autosome ratio of N_e in epoch h.
t	The scaled divergence time.
T_h	The duration of epoch h in generations.
u_k	The mutation rate per site per generation at locus k.
u_X and u_A	The mutation rate per site per generation on the X chromosome and autosomes, respectively.
$\boldsymbol{X}$	x_1, x_2, ..., x_K, where x_k is the observed number of substitutions at locus k.

Table S2: Mean of the MLEs for the parameters of Model 1 and Model 2. The simulations were conducted as described in Table 1 in the main text. Two divergence levels were considered, as specified by the parameters c and t.

	θ_X	θ_A	r_1	r_2	$g_{X,1}$	τ_1	c	Low divergence		High divergence	
								t	$c+t$	t	$c+t$
Model 1 (true)	5.25×10^{-4}	7.5×10^{-4}	0.65	0.75	10	0.1	2	10.67	12.67	77.33	79.33
No div	5.259×10^{-4}	7.53×10^{-4}	0.653	0.752	10.0	0.10	–	–	–	–	–
Low div	5.258×10^{-4}	7.52×10^{-4}	0.652	0.752	10.0	0.10	1.9	10.74	12.64	–	–
High div	5.230×10^{-4}	7.41×10^{-4}	0.638	0.741	10.0	0.10	3.6	–	–	76.07	79.65
Model 2 (true)	5.25×10^{-4}	7.5×10^{-4}	0.9	0.75	0.2	0.05	2	10.67	12.67	77.33	79.33
No div	5.261×10^{-4}	7.41×10^{-4}	0.89	0.742	0.20	0.051	–	–	–	–	–
Low div	5.270×10^{-4}	7.18×10^{-4}	0.87	0.720	0.20	0.051	2.9	9.77	12.63	–	–
High div	5.289×10^{-4}	7.18×10^{-4}	0.88	0.717	0.20	0.052	10.1	–	–	68.69	78.81

Table S3: The effects of very recent population expansion and limited sample size on parameter estimation. The population size increased instantly with parameter $g_{X,1} = 13$ (i.e. a 2-epoch demographic model). Several values of τ_1, the scaled time to the expansion event, were considered. For simplicity, we set the X-autosome ratio of N_e to 1 (i.e., $r_1 = r_2 = 1$). The scaled mutation rate are $\theta_X = 0.013$ and $\theta_A = 0.023$. As in the real dataset, the sample size is 21. The results are based on 100 replicates. Note that these results are likely to underestimate the uncertainties in estimating r_1 because the simulations assumed that all sites were unlinked.

τ_1 (true)	Mean(MLE)					
	θ_X	θ_A	r_1	r_2	$g_{X,1}$	τ_1
0.2	0.0130	0.0236	3.38	1.03	34.24	0.20
0.3	0.0129	0.0232	1.24	1.01	15.44	0.30

Figure S1: A two-locus model. This model considers X-autosome comparisons. The X chromosome is used as the reference locus. The free parameters include θ_X, θ_A, f, $g_{X,1}$, $g_{A,1}$, τ_1, where $\theta_X = 4N_X u_X$, $\theta_A = 4N_X u_A$, and $\tau_1 = T/(2N_X)$. The X-autosome ratio of N_e in epoch 1 is $r_1 = g_{X,1}/(f g_{A,1})$, and that in epoch 2 is $r_2 = 1/f$.

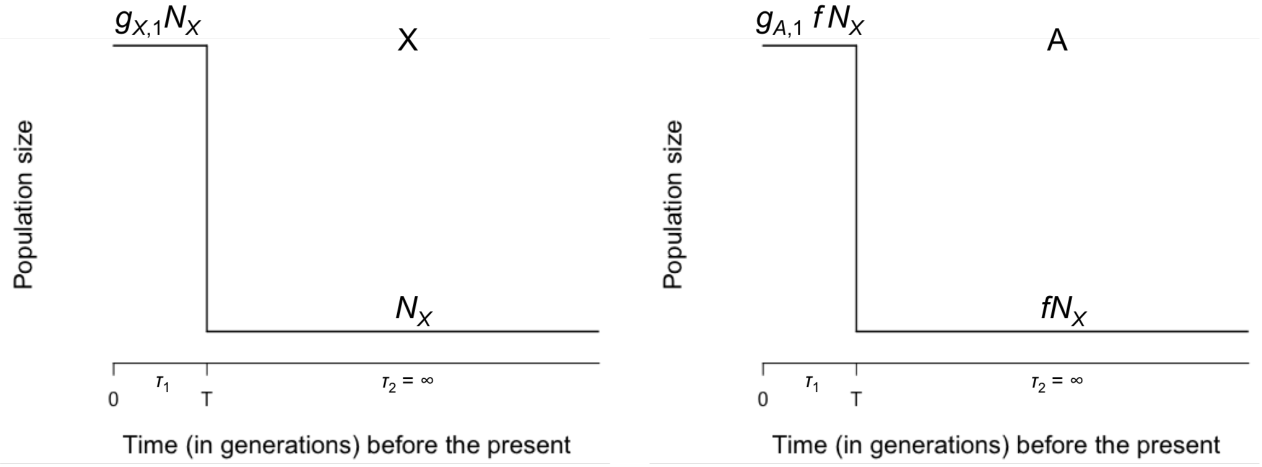

Figure S2: The effects of using locus 20 as the reference locus. The model is the same as that used in Figure 1, except that locus 20 was used as the reference. The true demographic parameters are $g_1 = 10$ and $\tau_1 = 0.1$ (τ_1 is 5 times smaller here because N_E at locus 2 is 5 times higher than that at locus 1). The mean (SD) of the MLEs of g_1 and τ_1 are 10.10 (0.29) and 0.10 (0.02) without divergence data, and 10.08 (0.29) and 0.10 (0.02) with divergence data. Note that locus 1, 2, 3, ... in the following plots correspond to locus 20, 19, 18, ... in Figure 1.

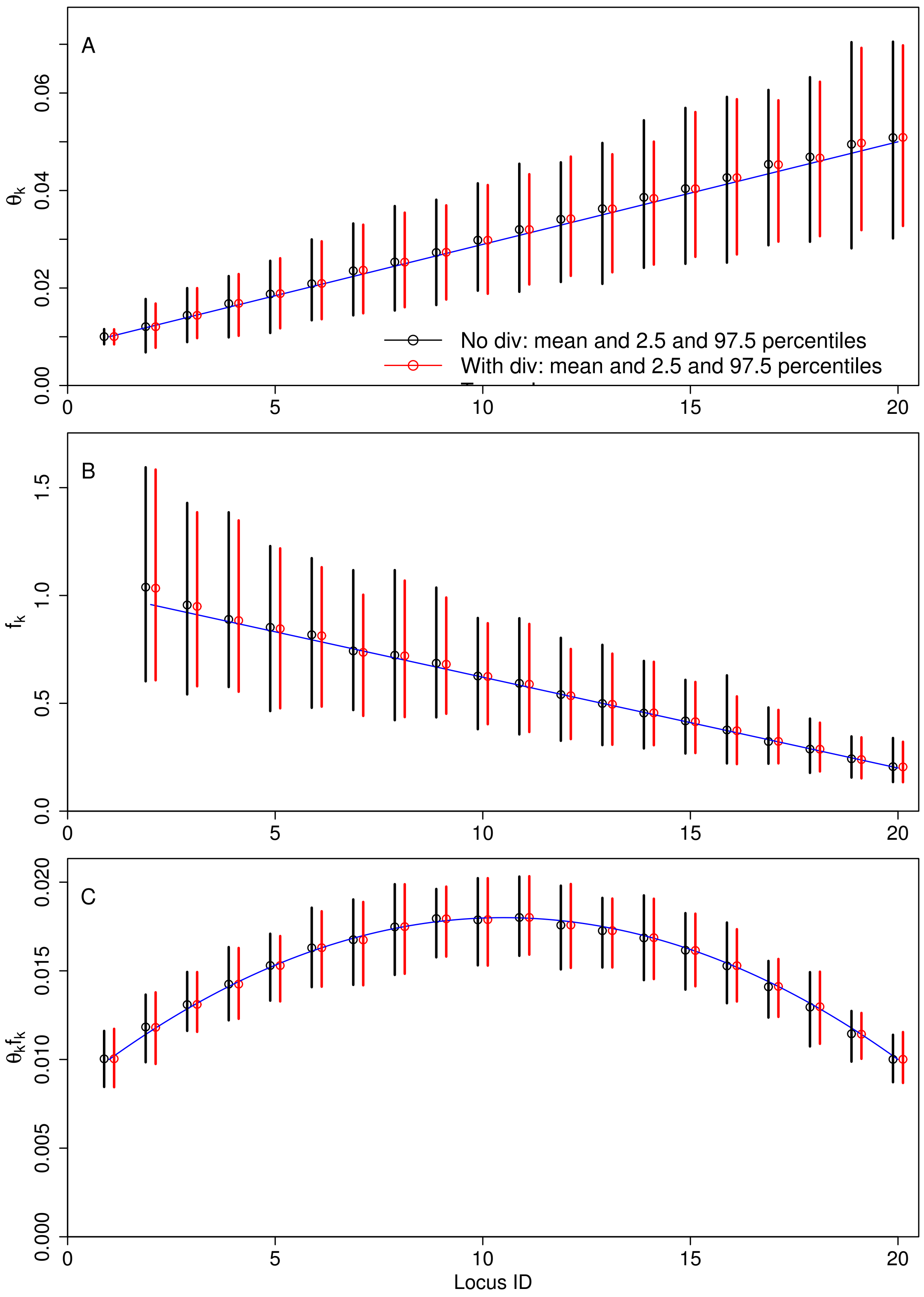

Figure S3: Comparing the observed uSFSs in *D. simulans* to those predicted by the no-error model with $H = 2$ or $H = 3$.

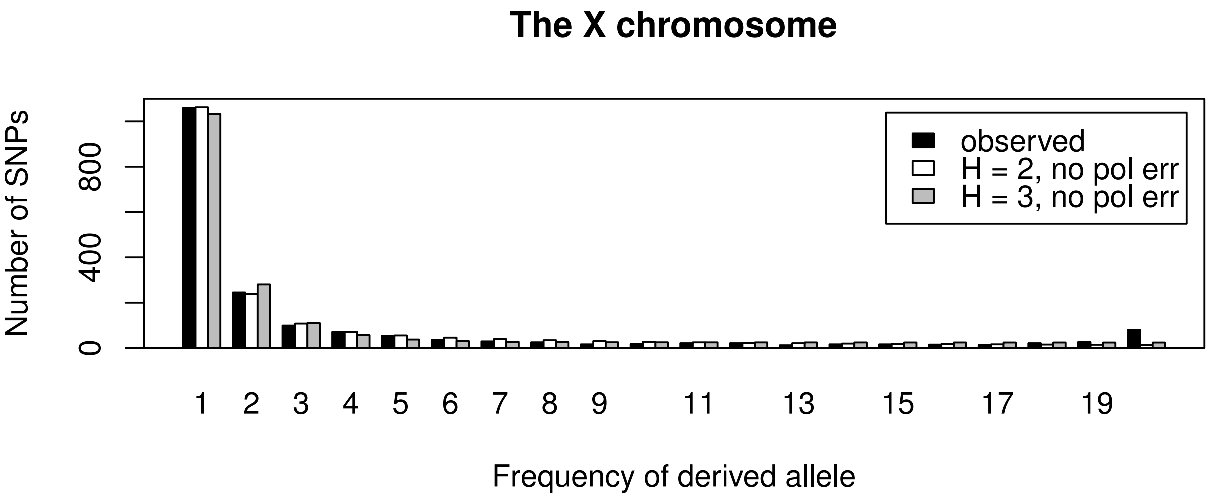

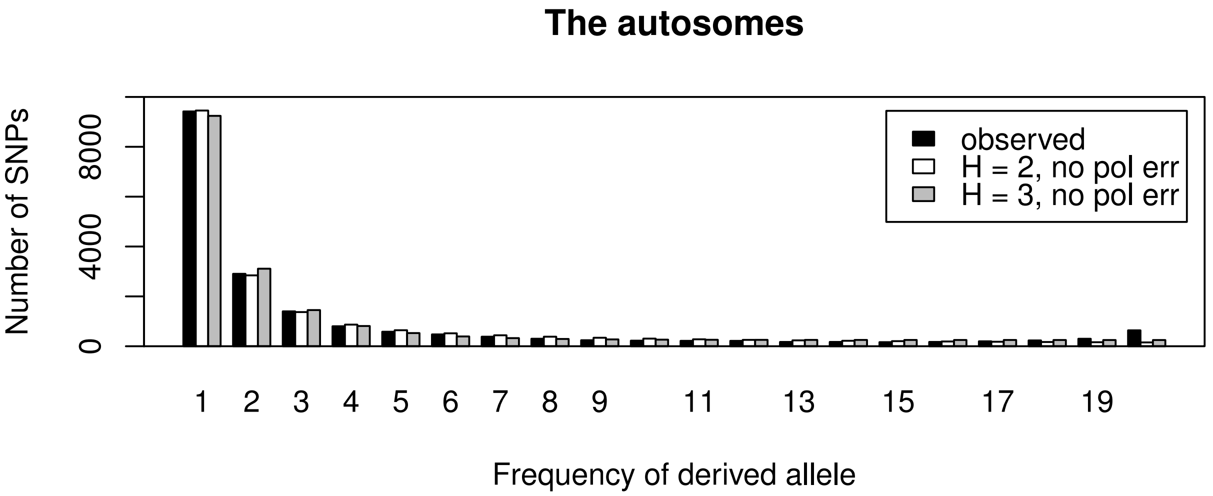

Figure S4: Comparing the observed uSFSs in *D. simulans* to those predicted by the model with $H = 2$ and polarisation error.

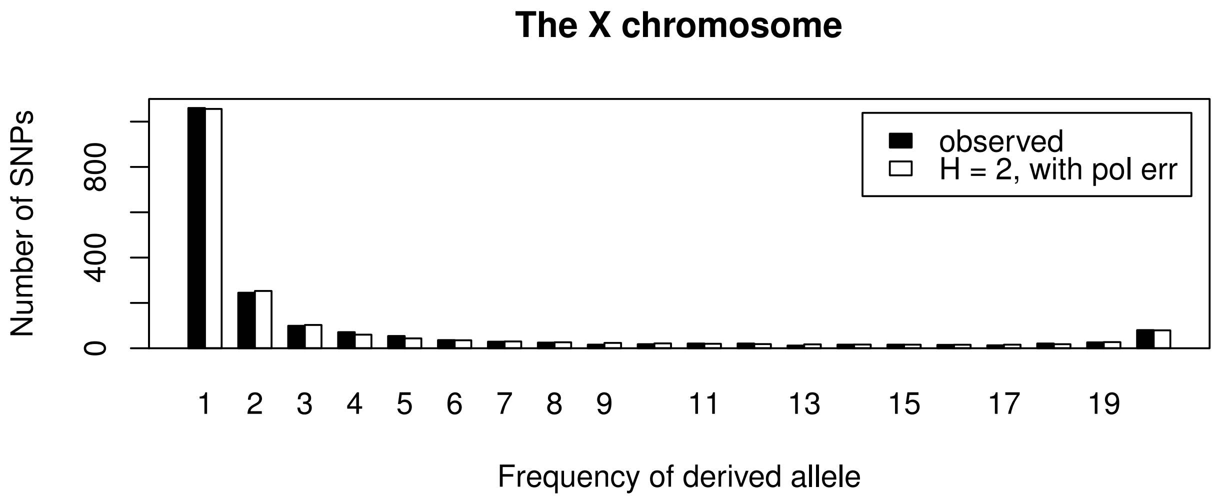

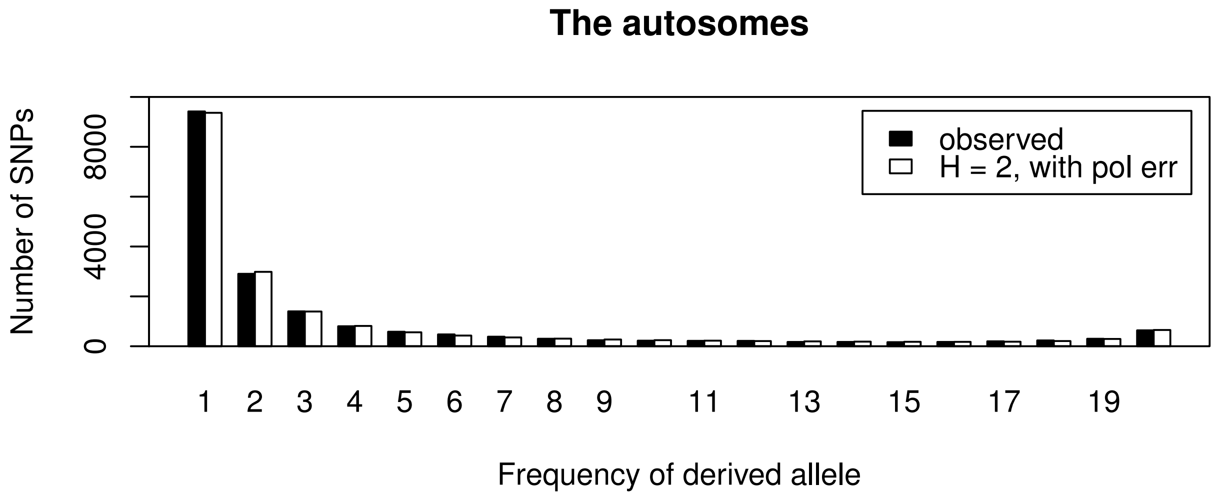

Supplement: Supplementary Data [file msy212_supp.zip › v3_rev_supp.pdf]
